# Mixture prior for sparse signals with dependent covariance structure

**DOI:** 10.1371/journal.pone.0284284

**Published:** 2023-04-27

**Authors:** Ling Wang, Zongqiang Liao

**Affiliations:** 1 Department of Medicine, College of Human Medicine, Michigan State University, East Lansing, Michigan, United States of America; 2 Institute for Health Policy, College of Human Medicine, Michigan State University, East Lansing, Michigan, United States of America; Dartmouth College, UNITED STATES

## Abstract

In this study, we propose an estimation method for normal mean problem that can have unknown sparsity as well as correlations in the signals. Our proposed method first decomposes arbitrary dependent covariance matrix of the observed signals into two parts: common dependence and weakly dependent error terms. By subtracting common dependence, the correlations among the signals are significantly weakened. It is practical for doing this because of the existence of sparsity. Then the sparsity is estimated using an empirical Bayesian method based on the likelihood of the signals with the common dependence removed. Using simulated examples that have moderate to high degrees of sparsity and different dependent structures in the signals, we demonstrate that the performance of our proposed algorithm is favorable compared to the existing method which assumes the signals are independent identically distributed. Furthermore, our approach is applied on the widely used “Hapmap” gene expressions data, and our results are consistent with the findings in other studies.

## Introduction

Feature selection in normal mean with high-dimensional data is one of main challenges in Genetics studies. The feature selection in normal mean problem can be described as a *p*-dimensional vector *Y* satisfying ***Y*** = ***X*** + ***Z***. In particular, ***X*** is a sparse vector, that is, a great portion of elements of ***X*** are zeros and the proportion of zeros in ***X*** is unknown. ***Z*** is a vector of noises and has a distribution as Z∼N(0,Σ), where **Σ** is the covariance matrix with arbitrary dependent structure. The purpose of this study is to find the desirable estimate of ***X*** vector which has the accurate sparsity information while considering correlations in the error vector ***Z***.

Traditionally, there are mainly two branches of studies in feature selection under normal mean issue. One branch of studies focuses primarily on constructing and selecting subset of features that are useful to build a good predictor where ***Y*** could be the regression coefficients, e.g. [1–4]. Another branch of studies uses multiple hypothesis testing in genomics/bio-informatics where test statistics could be considered as ***Y***, such as [5–7].

The study [8] was the first study that employed empirical Bayesian estimation method to find the sparsity in sparse vectors observed in Gaussian white noises. They assumed a mixture of point mass at zero and parametric distribution as the prior distribution for the sparse vectors, and inferred theoretical statistical properties for the estimators. [4] extended the work of [8] to allow more flexibility in the estimation by assuming the prior distribution as a mixture of point mass and nonparametric distribution. Nonetheless, both studies assumed that sparse signals in normal mean problem were independent identically distributed.

Another study [7] investigated the problem of controlling false discovery rate (FDR) with dependent structure in test statistics of multiple testing. They used an innovative principle factor approximation (PFA) procedure to significantly weaken the correlation structure. However, their study used a common p-value threshold to select the relevant features which can not be adapted to estimate sparsity in signals.

Our study extends the work of [4, 8] to allow sparse signals to be correlated and have arbitrary covariance structure, and adopts the PFA procedure of [7] to estimate sparsity in signals automatically by assuming a mixture prior in an empirical Bayesian estimating setup. The motivation for allowing correlation in sparse signals comes from gene expressions data analyses that need to identify a proportion of genes associated with the outcome. There are usually statistical correlations between genes in gene expression data because of either biological reason (i.e. some genes are connected in biochemical pathways, [9]), or technical reason (i.e. imperfect normalization, [10]). Ignoring the correlation between genes may cause high variability of statistical estimators and even compromise their consistency [6, 11]. Furthermore, there exists a sparsity problem: although the number of genes is large, there are maybe only a very small subset of genes that contribute to the outcome, [7].

To find the sparsity in a high-dimensional sparse vector with correlations, we first apply spectral decomposition on the covariance matrix of the error terms and take out the principle factors that derive the strong dependence in the covariance matrix so that the remaining factors have weak dependence. Because of the existence of sparsity, the approximation is accurate [7]. Then, we employ an empirical Bayesian method to estimate the sparsity based on the marginal likelihood of signals without strong dependence. We consider a num-ber of dependent structures in the sparse signals for simulation studies. The results demon-strate the advantages of our empirical Bayesian (DepEB) estimator considering dependence over the empirical Bayesian (EB) estimator proposed by [4] without considering depen-dence when there exists strong dependence in the covariance: 1) Our DepEB estimator pro-vides more accurate estimates of the sparsity than the EB estimator; 2) Our DepEB estimator gives smaller (integrated) mean squared errors (MSEs) than the EB estimator. In a real gene data application, we consider observed signals as the corresponding marginal regression coefficients of the genes on the outcome. There are strong correlations among the marginal coefficients because of the high association between genes. Our proposed DepEB estimator outperforms the EB estimator, and our results are also consistent with other studies.

In sum, our estimating method has two main features. First, our estimating method allows the covariance of the observed high-dimensional signals to have arbitrary dependent structure instead of assuming the signal vector follows an independent and identically distribution. Second, our estimating method incorporates the possibility of sparsity and uses a mixture prior with an atom of probability at zero and a non-parametric density for the nonzero part. We treat the mixture probability as well as the non-parametric part as hyperparameters, and we estimate them by an empirical Bayesian procedure automatically.

The remainder of this paper is organized as follows. Section *Materials and Methods* explicitly describes our model and estimation algorithm for our proposed estimator. In section *Simulation Studies*, the performance of the proposed estimator is evaluated by a number of simulation studies. Section *Real Data Analysis* presents the real data analysis using our proposed estimator. Section *Conclusion* concludes the paper.

## Materials and methods

### Model

We are interested in estimating measurement error models where we observe only the error-contaminated variable *Y*_*i*_ in the equation:

$$\begin{array}{c} Y_{i}=X_{i}+Z_{i},\;i=1,\ldots ,p, \end{array}$$ (1)

where *Z*_1_, …, *Z*_*p*_ are measurement errors with joint distribution as N(0,Σ). This study allows arbitrary dependent structure in the covariance matrix **Σ**. *X*_*i*_ is assumed to come from a mixture of a delta function with point mass at zero and a completely unspecified nonparametric density *θ*

$$\begin{array}{c} P\left(X_{i}|\psi ,\theta \right)=\psi \delta \left(X_{i}\right)+\left(1-\psi \right)\theta \left(X_{i}\right), \end{array}$$ (2)

where *δ*(*X*_*i*_) is the Dirac delta function when *X*_*i*_ = 0 and *ψ* ∈ [0, 1] describes the prior probability when *X*_*i*_ = 0. This prior structure represents our belief that some of *X*_1_, …, *X*_*p*_ are exactly zero, i.e. the mixing parameter *ψ* is the fraction of zeros in *X*_1_, …, *X*_*p*_ corresponding to the number of irrelevant features. We treat *ψ* as a hyperparameter and estimate it using an empirical Bayesian approach. For the nonzero part of the prior *θ*(*X*_*i*_), there were a number of parametric distribution prior specifications used by previous studies, such as Laplace distribution in [12], Student-*t* distribution in [13], and normal distribution in [14]. Although these previous studies have shown that the parametric specifications are successful, they all need to assume a specific shape for the nonzero part. This study takes a more realistic approach by leaving the nonzero part of the prior, *θ*(*X*_*i*_), completely unspecified as proposed in [4].

### Principal factor approximation

Based on the observation of *Y*_1_, …, *Y*_*p*_ in Eq 1, we estimate the values for *X*_1_, …, *X*_*p*_. In the sparse scenario, estimated $\hat{X_{i}}$ need to find the correct degree of sparsity in *X*_*i*_.

Because covariance matrix **Σ** of *Z*_1_, …, *Z*_*p*_ can have arbitrary dependent structure, the first step of estimation is to remove dependence among the error terms *Z*_*i*_. Therefore, we approximate the likelihood function of signals *Y*_1_, …, *Y*_*p*_ with weakly dependent normal random variables as proposed in [7].

The definition of weakly dependent normal random variables is stated as

**Definition 1** If a set of random variables (*I*_1_, …, *I*_*p*_)^*T*^ has the normal distribution $N({\left(\mu_{1},\ldots ,\mu_{p}\right)}^{T},C)$ and the (*i*, *j*)th element *c*_*ij*_ in the covariance matrix *C* satisfying the condition

$$\begin{array}{c} \lim_{p\to \infty }\frac{\sum_{i,j}\left|c_{ij}\right|}{p^{2}}=0, \end{array}$$ (3)

then *I*_1_, …, *I*_*p*_ are called weakly dependent normal random variables.

We apply principal factor approximation (PFA) technique in [7] to decompose the covariance matrix **Σ** so Eq 1 becomes a factor model with weakly dependent normal random errors. Before PFA procedure, the estimate of **Σ** need to be calculated since in real data set, true **Σ** is usually unknown. We propose to use sample covariance of the errors as the estimate for **Σ**.

The details of PFA procedure are described in the following steps:

1. Apply spectral decomposition on the covariance matrix **Σ**. Denote eigenvalues of **Σ** as *λ*_1_, …, *λ*_*p*_, which are arranged from the largest to the smallest. Denote the corresponding eigenvectors as *γ*_1_, …, *γ*_*p*_, then $\Sigma =\sum_{i=1}^{p}\lambda_{i}\gamma_{i}\gamma_{i}^{T}$.
2. Further separate $\Sigma =\sum_{i=1}^{p}\lambda_{i}\gamma_{i}\gamma_{i}^{T}$ to two parts for an appropriate integer *k* so $\Sigma =\sum_{i=1}^{k}\lambda_{i}\gamma_{i}\gamma_{i}^{T}+A$, where $A=\sum_{i=k+1}^{p}\lambda_{i}\gamma_{i}\gamma_{i}^{T}$ and *k* is chosen as the smallest *k* such that
  $$\begin{array}{c} \frac{\sqrt{\lambda_{k+1}^{2}+...+\lambda_{p}^{2}}}{\lambda_{1}+...+\lambda_{p}}\leq \epsilon \end{array}$$ (4)
  for a predetermined small value *ϵ*, say 0.05. Let $L=(\sqrt{\lambda_{1}}\gamma_{1},\ldots ,\sqrt{\lambda_{k}}\gamma_{k})$. Then the covariance matrix **Σ** can be decomposed as **Σ** = *LL*^*T*^ + *A*.
3. Rewrite Eq 1 as $Y_{i}=X_{i}+\sum_{h=1}^{k}b_{ih}W_{h}+K_{i}$, *i* = 1, …, *p*. Here ${\left(b_{1j},\ldots ,b_{pj}\right)}^{T}=\sqrt{\lambda_{j}}\gamma_{j}$ for *j* = 1, …, *k*. Each factor $W_{h}∼N\left(0,1\right)$ for *h* = 1, …, *k* and the random errors ${\left(K_{1},\ldots ,K_{p}\right)}^{T}∼N\left(0,A\right)$. As shown in [7], *K*_1_, …, *K*_*p*_ are weakly dependent normal random variables based on *k* chosen by Eq 4.
4. Estimate factors *W*_1_, …, *W*_*k*_ based on the data. From observed values *Y*_1_, …, *Y*_*p*_, choose the first *s* *Y*_*i*_’s that have the smallest 90% percentile absolute values. Then approximately, $Y_{i}=\sum_{h=1}^{k}b_{ih}W_{h}+K_{i},i=1,\ldots ,s$ because the existence of sparsity makes this approximation practical. $\hat{W_{1}},...,\hat{W_{k}}$ are obtained by least-absolute deviation regression on the approximation equation.

After applying PFA procedures on the covariance matrix **Σ**, we can re-write the normal mean problem with dependent error terms in Eq 1 as

$$\begin{array}{c} Y_{i}=X_{i}+\sum_{h=1}^{k}b_{ih}\hat{W_{h}}+K_{i},\;i=1,\ldots ,p. \end{array}$$ (5)

Denote $\phi_{i}=Y_{i}-\sum_{h=1}^{k}b_{ih}\hat{W_{h}}$, then Eq 5 can be rewritten as

$$\begin{array}{c} \varphi_{i}=X_{i}+K_{i},\;i=1,\ldots ,p, \end{array}$$ (6)

where *K*_*i*_’s are weakly dependent normal variables with distribution as $N(0,a_{i}^{2})$ and $a_{i}=\sqrt{\text{Var}\left(Z_{i}\right)-\sum_{h=1}^{k}b_{ih}^{2}}$ for *i* = 1, …, *p*.

### Empirical bayesian estimation

Here, we estimate the hyperparameter $\hat{\psi }$ in Eq 2 using empirical Bayesian method.

#### Posterior distribution

We first derive the posterior distribution based on the likelihood function $P(\phi_{i}|X_{i})$ and the prior distribution of *X*_*i*_, for *i* = 1, …, *p*.

From Eq 6 we have $P\left(\phi_{i}|X_{i}\right)=N\left(\phi_{i}|X_{i},a_{i}\right)$. Since *K*_*i*_’s are weakly dependent, the likelihood of the parameters ***ϕ*** given the observations ***X*** can be approximated as

$$\begin{array}{c} P\left(\phi |X\right)\approx \prod_{i=1}^{p}P\left(\phi_{i}|X_{i}\right)=\prod_{i=1}^{p}N\left(\phi_{i}|X_{i},a_{i}\right). \end{array}$$ (7)

Given the prior distribution of ***X*** in Eq 2, i.e. $P(X_{i}|\psi ,\theta )$ and likelihood P(ϕ|X), the posterior of ***X*** given ***ϕ*** can be written as

$$\begin{array}{c} P\left(X|\phi ,\psi ,\theta \right)=\frac{\prod_{i=1}^{p}P\left(\phi_{i}|X_{i}\right)P\left(X_{i}|\psi ,\theta \right)}{m(\phi |\psi ,\theta )}, \end{array}$$ (8)

where *m*(***ϕ***|*ψ*, *θ*) is the marginal of the data given the hyper-parameter *ψ* and the nonparametric distribution *θ*

$$\begin{array}{c} m\left(\phi |\psi ,\theta \right)=\prod_{i=1}^{p}\int P\left(\phi_{i}|X_{i}\right)P\left(X_{i}|\psi ,\theta \right)dX_{i}. \end{array}$$ (9)

Replace $P(\phi_{i}|X_{i})$ by $N(\phi_{i}|X_{i},a_{i})$ and substitute $P(X_{i}|\psi ,\theta )$ by Eq 2, then Eq (9) can be rewritten as

$$\begin{array}{ccc} m(\phi |\psi ,\theta ) & = & \prod_{i=1}^{p}\int N\left(\phi_{i}|X_{i},a_{i}\right)\left[\psi \delta \left(X_{i}\right)+\left(1-\psi \right)\theta \left(X_{i}\right)\right]dX_{i}. \end{array}$$

That is

$$\begin{array}{ccc} m(\phi_{i}|\psi ,\theta ) & = & \psi N\left(\phi_{i}|\sum_{h=1}^{k}b_{ih}\hat{W_{h}},a_{i}\right)+\left(1-\psi \right)\int N\left(\phi_{i}|X_{i},a_{i}\right)\theta \left(X_{i}\right)dX_{i}\\ & = & \psi N\left(\phi_{i}|\sum_{h=1}^{k}b_{ih}\hat{W_{h}},a_{i}\right)+\left(1-\psi \right)g\left(\phi_{i}\right), \end{array}$$ (10)

where

$$g\left(\phi_{i}\right)=\int N\left(\phi_{i}|X_{i},a_{i}\right)\theta \left(X_{i}\right)dX_{i}.$$

Then the posterior of *X*_*i*_ can be written as:

$$P\left(X_{i}|\phi_{i},\psi ,\theta \right)=\frac{\psi \delta \left(X_{i}\right)N\left(\phi_{i}|\sum_{h=1}^{k}b_{ih}\hat{W_{h}},a_{i}\right)+\left(1-\psi \right)\theta \left(X_{i}\right)N\left(\phi_{i}|X_{i},a_{i}\right)}{\psi N\left(\phi_{i}|\sum_{h=1}^{k}b_{ih}\hat{W_{h}},a_{i}\right)+\left(1-\psi \right)g\left(\phi_{i}\right)}.$$

Define

$$q_{i}=\frac{\psi N(\phi_{i}|\sum_{h=1}^{k}b_{ih}\hat{W_{h}},a_{i})}{\psi N\left(\phi_{i}|\sum_{h=1}^{k}b_{ih}\hat{W_{h}},a_{i}\right)+\left(1-\psi \right)g\left(\phi_{i}\right)}$$

and

$$G\left(X_{i}\right)=\frac{N\left(X_{i}|\phi_{i},a_{i}\right)\theta \left(X_{i}\right)}{\int N\left(X_{i}|\phi_{i},a_{i}\right)\theta \left(X_{i}\right)dX_{i}},$$

then

$$\begin{array}{ccc} P(X_{i}|\phi_{i},\psi ,\theta ) & = & q_{i}\delta \left(X_{i}\right)+\left(1-q_{i}\right)G\left(X_{i}\right). \end{array}$$ (11)

Notice that *q*_*i*_ is the posterior density of *X*_*i*_ when it is 0 and *G*(*X*_*i*_) is the posterior density of *X*_*i*_ when it is not 0. That is

$$\begin{array}{ccc} P(X_{i}=0|\phi_{i},\psi ,\theta ) & = & q_{i},\\ P(X_{i}|\phi_{i},\psi ,\theta ,X_{i}\neq 0) & = & G(X_{i}). \end{array}$$

#### Estimate the hyperparameter *ψ*

We use an empirical Bayesian approach in [15] to estimate the hyperparameter *ψ* by maximizing the marginal likelihood *m*(***ϕ***|*ψ*, *θ*)

$$\begin{array}{ccc} \hat{\psi } & = & \text{arg}\underset{\psi }{\text{max}}m\left(\phi |\psi ,\theta \right)=\text{arg}\underset{\psi }{\text{max}}\text{log}m\left(\phi |\psi ,\theta \right)\\ & = & \text{arg}\underset{\psi }{\text{max}}\sum_{i=1}^{p}\text{log}\left[\psi N\left(\phi_{i}|\sum_{h=1}^{k}b_{ih}\hat{W_{h}},a_{i}\right)+\left(1-\psi \right)g\left(\phi_{i}\right)\right]. \end{array}$$ (12)

Notice $g\left(\phi_{i}\right)=\int N\left(\phi_{i}|X_{i},a_{i}\right)\theta \left(X_{i}\right)dX_{i}$, which is the marginal density of non-zero *X*_*i*_’s. It is proposed by [4] to estimate *g*(*ϕ*_*i*_) directly. In this way, we do not have to specify any prior distribution form for the non-zero part of *X*_*i*_’s. We use a weighted non-parametric kernel density estimator in [4] to estimate *g* of the following form

$$g\left(\phi_{i}\right)=\frac{1}{p\nu }\sum_{j=1}^{p}\left(1-q_{i}\right)R(\frac{\phi_{i}-\phi_{j}}{\nu }),$$

where *R* is a kernel function satisfying ∫*R*(*x*)*dx* = 1 and *ν* is a positive number called band-width of the kernel. The most widely used kernel is a normal density with zero mean and unit variance, that is, R(x)=N(x|0,1). Therefore, we set the bandwidth of the kernel using the normal reference rule $\nu =O(p^{-1/5})$ as in [16].

In summary, the hyperparameter *ψ* can be estimated by the following iterative steps

Algorithm

1. Given the current estimate $\hat{g}\left(\phi_{i}\right)$, obtain $\hat{\psi }$ by maximizing the log-marginal in Eq (12) numerically.

2. Compute $\hat{q_{i}}$ using the current estimated $\hat{\psi }$ and $\hat{g}\left(\phi_{i}\right)$

$$\hat{q_{i}}=\frac{\hat{\psi }N\left(\phi_{i}|\sum_{h=1}^{k}b_{ih}\hat{W_{h}},a_{i}\right)}{\hat{\psi }N\left(\phi_{i}|\sum_{h=1}^{k}b_{ih}\hat{W_{h}},a_{i}\right)+\left(1-\hat{\psi }\right)\hat{g}\left(\phi_{i}\right)}.$$

3. Re-estimate $\hat{g}\left(\phi_{i}\right)$ using the current estimate of $\hat{q_{i}}$

$$\begin{array}{c} \hat{g}\left(\phi_{i}\right)=\frac{1}{p\nu }\sum_{j=1}^{p}\left(1-\hat{q_{i}}\right)R(\frac{\phi_{i}-\phi_{j}}{\nu }). \end{array}$$ (13)

#### Posterior mean

We use the mean of the posterior as a point estimate for non-zero part of *X*_*i*_’s.

$$\begin{array}{c} {\hat{X}}_{i}=\left(1-\hat{q_{i}}\right)E_{G}\left[X_{i}\right], \end{array}$$ (14)

where

$$E_{G}\left[X_{i}\right]=\frac{\int X_{i}N\left(X_{i}|\phi_{i},a_{i}\right)\theta \left(X_{i}\right)dX_{i}}{\int N\left(X_{i}|\phi_{i},a_{i}\right)\theta \left(X_{i}\right)dX_{i}}=\phi_{i}+{\hat{g}}^{\prime }\left(\phi_{i}\right)/\hat{g}\left(\phi_{i}\right),$$

and the estimate of marginal $\hat{g}\left(\phi_{i}\right)$ is obtained using Eq 13 and the estimate of derivative ${\hat{g}}^{\prime }\left(\phi_{i}\right)$ is given by

$${\hat{g}}^{\prime }\left(\phi_{i}\right)=\frac{1}{p\nu^{2}}\sum_{j=1}^{p}\left(1-\hat{q_{i}}\right)R^{\prime }(\frac{\phi_{i}-\phi_{j}}{\nu }).$$

## Simulation studies

In this section, we conduct simulation studies to evaluate our proposed estimation procedure and make comparisons between our method with the one in [4]. We simulate the data for Eq 1. A sequence of *p* = 1000 signals *Y*_*i*_’s are generated with 200 repetitions in the simulation study.

We generate *X*_*i*_ using a type of non-zero distribution with different degree of sparsity. Two different types of distribution are considered for the non-zero values in *X*_*i*_’s: 1) Uninormal and 2) Binormal. The detailed descriptions for the distribution of *X*_*i*_’s used in the simulation studies are summarized in Table 1. We let *X*_*i*_ equal to zero at randomly selected positions and the proportion of zeros in *X*_*i*_’ ranges from 0.6 to 0.9 with interval of 0.1, which represents the situation from low sparsity to high sparsity. Because the existence of sparsity makes the PFA approximation practical as specified in Eq 5, we consider relatively high values of sparsity parameter *ψ* in the simulation studies.

**Table 1:** Distribution types of *X*_*i*_’s for different sparsity of *ψ*.

Uninormal (Unimodal Normal)	$\begin{array}{c} X_{i}=\{\begin{array}{cc} 0, & \text{if}\;V_{i}=0,\\ N(5,1), & \text{if}\;V_{i}=1, \end{array}\\ \;\;\;\;\text{and}\;\;P(V_{i}=0)=\psi \text{,}\;\;\psi =0.6,\ldots ,0.9 \end{array}$
Binormal (Bimodal Normal)	$\begin{array}{c} X_{i}=\{\begin{array}{cc} 0, & \text{if}\;V_{i}=0,\\ \;N(3,1)\;\;\text{with}\;P=0.4\\ \text{or} & \text{if}\;V_{i}=1,\\ N(8,1)\;\;\text{with}\;P=0.6, \end{array}\\ \;\;\;\;\text{and}\;\;P(V_{i}=0)=\psi \text{,}\;\psi =0.6,\ldots ,0.9 \end{array}$

The observed value of *Y*_*i*_ is generated by adding noise *Z*_*i*_ for each *X*_*i*_ based on Eq 1. Let Z∼N(0,Σ). Our simulation studies consider six different dependent structures in the covariance of noises ***Z***. The dependent structures of **Σ** are similar to the settings used in [7], and they are generated using random variables ${\left\{B_{i}\right\}}_{i=1}^{p}$ with a sample size of *n* = 100. The covariance matrix **Σ** is the sample correlation matrix of *B*_1_, ⋯, *B*_*p*_.

1. **Independent Cauchy**: let *B*_*i*_ be i.i.d. Cauchy random variables with location parameter 0 and scale parameter 1.
2. **Three-Factor Model**: let $B_{i}=\rho_{i}^{\left(1\right)}D^{\left(1\right)}+\rho_{i}^{\left(2\right)}D^{\left(2\right)}+\rho_{i}^{\left(3\right)}D^{\left(3\right)}+H_{i}$, where $\rho_{i}^{\left(1\right)}$, $\rho_{i}^{\left(2\right)}$ and $\rho_{i}^{\left(3\right)}$ are i.i.d. U(-1,1), $D^{\left(1\right)}∼N\left(-2,1\right)$, $D^{\left(2\right)}∼N\left(1,1\right)$ and $D^{\left(3\right)}∼N\left(4,1\right)$, and *H*_*i*_ are i.i.d. N(0,1).
3. **Nonlinear-Factor Model**: let $B_{i}=\text{sin}\left(\rho_{i}^{\left(1\right)}D^{\left(1\right)}\right)+\text{sgn}\left(\rho_{i}^{\left(2\right)}\right)\text{exp}(|\rho_{i}^{\left(2\right)}\left|D^{\left(2\right)}\right)+H_{i},i=1,\ldots ,1000$, where $\rho_{i}^{\left(1\right)}$ and $\rho_{i}^{\left(2\right)}$ are i.i.d. U(-1,1), *D*^(1)^ and *D*^(2)^ are i.i.d. N(0,1), and *H*_*i*_ are i.i.d. N(0,1).
4. **Fan & Song’s Model**: let ${\left\{B_{i}\right\}}_{i=1}^{900}$ be i.i.d. N(0,1), and $B_{i}=\sum_{l=1}^{10}Z_{l}{\left(-1\right)}^{l+1}/5+\sqrt{1-10/25}\eta_{i},i=901,\ldots ,1000$, where *η*_*i*_ for *i* = 901, …, 1000 are standard normally distributed.
5. **Equal Correlation of 0.4**: let diagonal elements of covariance matrix **Σ** be 1 and off-diagonal elements be 0.4 representing the strength of correlation in the error terms ***Z***.
6. **Equal Correlation of 0.8**: let diagonal elements of covariance matrix **Σ** be 1 and off-diagonal elements be 0.8.

**Fig 1:**
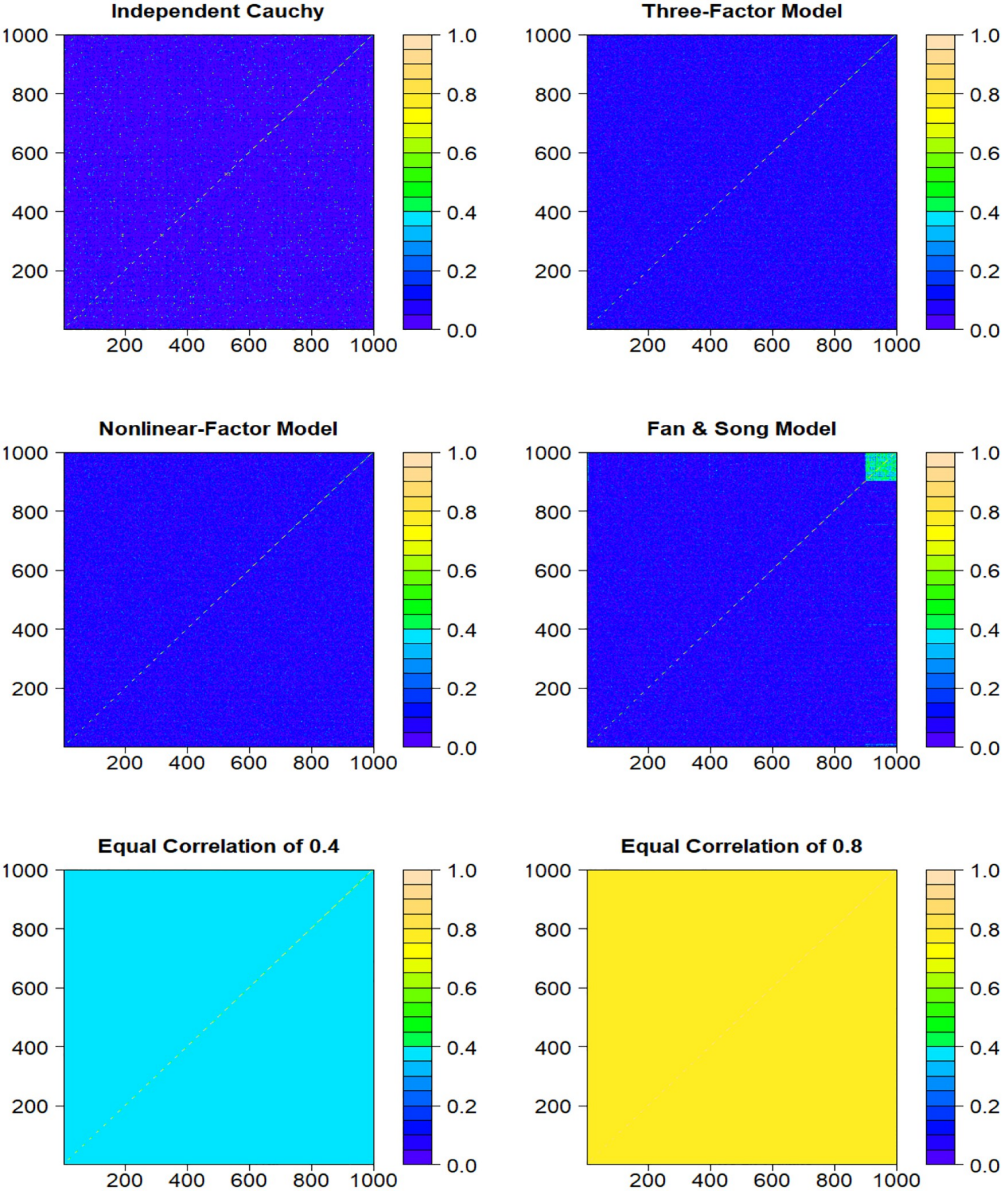
Heatmaps for six covariance structures of error terms *Z*.

The heatmaps for six covariance structures of error terms ***Z*** are shown in Fig 1. It can be seen from Fig 1 that the correlations of error terms in Independent Cauchy, Three-Factor Model, Nonlinear-Factor Model are very small. Fan & Song’s Model is very close to independent distribution but it has some dependence in a small part of the data points. The correlations of error terms in Equal Correlation of 0.4 and 0.8 models are much stronger than other covariance structures.

**Fig 2:**
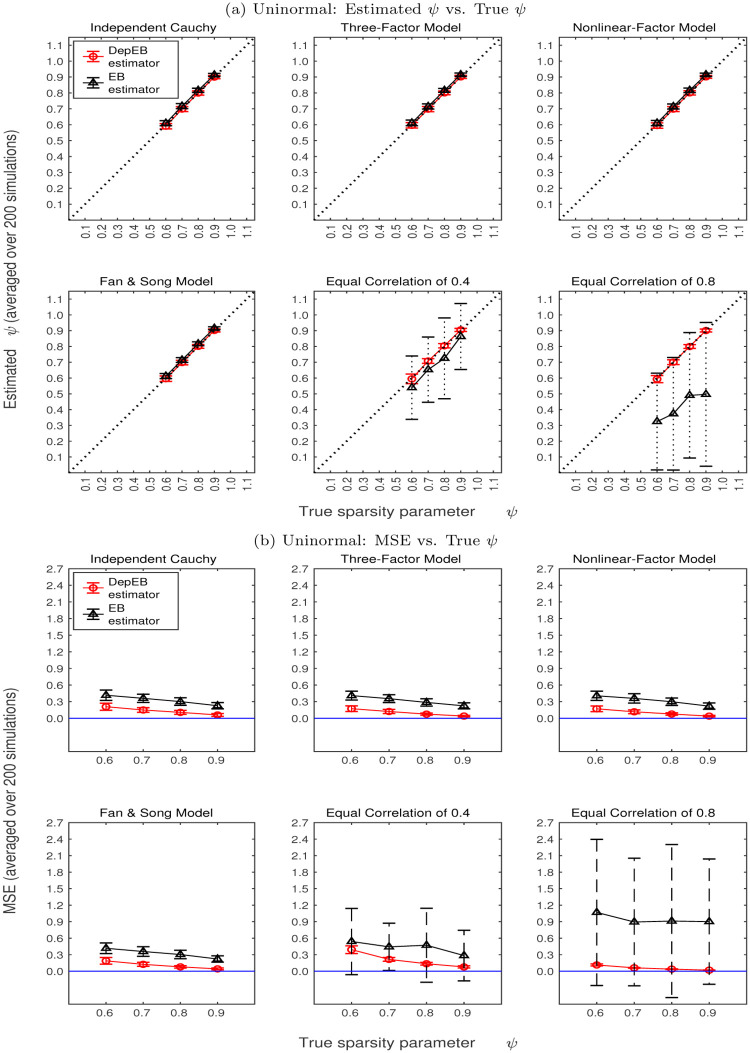
Estimated *ψ* and MSE for uninormal type of *X* and six covariance structures of error terms *Z*.

**Fig 3:**
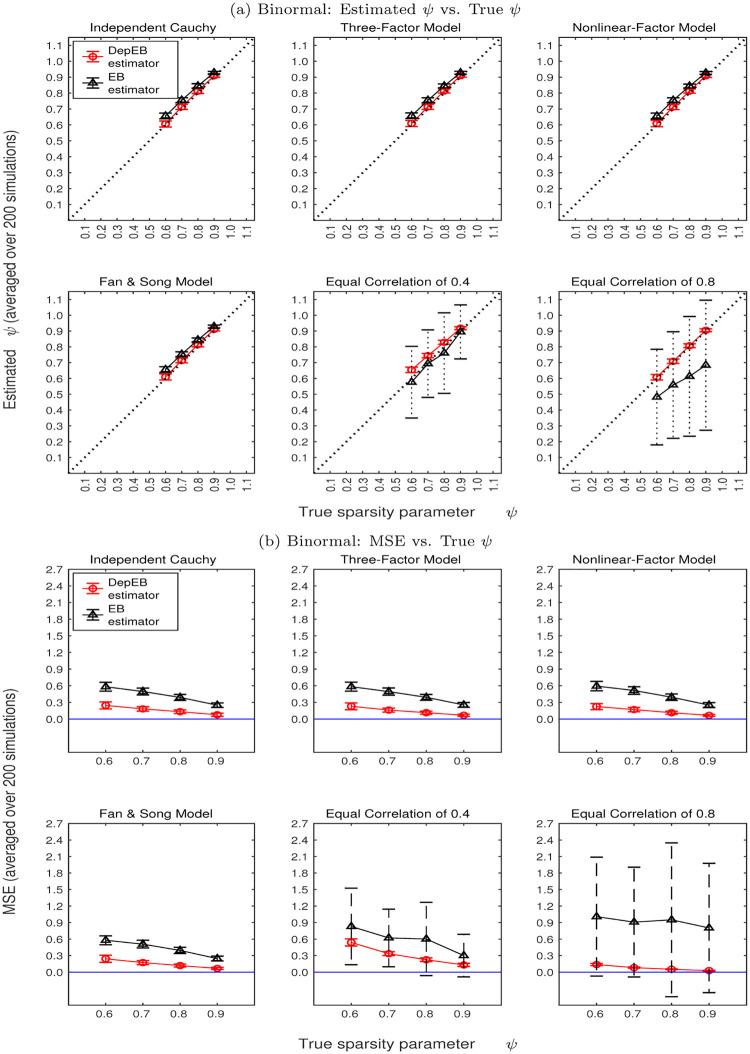
Estimated *ψ* and MSE for binormal type of *X* and six covariance structures of error terms *Z*.

Given ***Z***, we estimate the mixture probability of *ψ* and recover the posterior distribution of ***X***. Using simulated data sets, Figs 2 and 3 show estimated *ψ* and mean squared error (MSE) for Uninormal and Binormal types of ***X*** and six covariance structures of error terms *Z* over 200 simulations. To make comparison, Figs 2 and 3 show results obtained both by using our proposed DepEB estimation algorithm considering dependence in error terms (in red color) and the EB estimator proposed by [4] without considering dependence in error terms (in gray color).

Specifically, panels (a) in Figs 2 and 3 show means and standard deviations of estimated *ψ* over 200 repetitions versus true values of *ψ* when distribution of ***X*** follows Uninormal and Binormal, respectively. The first four sub-plots in panels (a) of Figs 2 and 3 show the estimated mixture probability *ψ* when the error terms are generated using Independent Cauchy Model, Three-Factor Model, Nonlinear-Factor Model and Fan & Song’s Model. Notice that the correlation structures of the error terms in these four models are very close to independent structure. In these cases, the results obtained by using our proposed DepEB estimation algorithm (in red color) and the EB estimator (in gray color) both can give estimates close to the true values of *ψ* and give small standard deviations of the estimates.

The last two sub-plots in panels (a) of Figs 2 and 3 show the estimated results when the error terms are generated using Equal Correlation of 0.4 and 0.8 structures, respectively. As shown in the heatmap Fig 1, these two types of dependence structure have much stronger dependence among error terms than the previous four dependence structures. It can be seen that our proposed DepEB estimators give quite accurate estimates and small standard deviations (in red color), which indicates our estimation algorithm can adapt quite well to high correlation in error terms. The distribution of error terms is clearly mis-specified if ignoring the dependence structure. Therefore, the EB estimators tend to underestimate *ψ* and give large standard deviations (in gray color), which indicates that the proportion of nonzeros would be overestimated and existence of big bias.

To evaluate the accuracy of the estimation for the non-zero part of ***X***, we calculate mean squared error $\text{MSE}=1/p\sum_{i=1}^{p}{\left(\hat{X_{i}}-X_{i}\right)}^{2}$, where $\hat{X}$ is the mean of posterior. Panels (b) in Figs 2 and 3 plot the means and standard deviations of MSE over 200 repetitions versus the true values of *ψ* when distribution of ***X*** follows Uninormal and Binormal, respectively.

The following findings can be seen from panels (b) in Figs 2 and 3: 1) MSEs estimated by our DepEB algorithm (in red color) are always lower than those estimated by the EB algorithm (in gray color). When correlations in error terms are very strong (e.g. Equal Correlation of 0.8 model), MSEs estimated by our proposed DepEB algorithm are much lower than those estimated by the EB algorithm; 2) As the signal becomes more sparse, the MSEs estimated by both the DepEB algorithm and EB algorithm become lower; 3) Similar to the findings in standard deviation of estimated *ψ*, standard deviations of MSEs in our proposed DepEB algorithm are small for different types of correlation structure in the error terms, while the standard deviations of MSEs estimated by the EB algorithm are large when there are moderate to strong correlations in the error terms (e.g. Equal Correlation of 0.4 and 0.8 models); 4) As the signal becomes more sparse, the standard deviations of MSEs estimated by both the DepEB algorithm and EB algorithm generally become smaller.

## Real data analysis

We use gene expression data set to test the validity of our proposed DepEB estimation algorithm. The gene expression data used in this study are for 90 unrelated Asians from the international “HapMap” project [17], which include 45 Japanese in Tokyo, Janpan (JPT) and 45 Han Chinese in Beijing, China (CHB). The gene expression data were generated by an Illumina Sentrix Human-6 Expression BeadChip [18] and have been normalized by using the ithquantile normalization across replicates and median normalization across individuals. These gene expression data have been used in [19, 20], and they are available on ftp://ftp.sanger.ac.uk/pub/genevar/ or https://doi.org/10.6084/m9.figshare.22491772.

### Distribution of marginal gene effects

We consider a outcome of interest *ζ* and gene data stored in a *n* × *p* matrix *S*. The gene data represent *p* gene expressions for *n* individuals. More specifically, element *S*_*ij*_ in *S* represents the *j*^*th*^ gene of the *i*^*th*^ individual. With the data, we perform marginal linear regression between the outcome variable *ζ* and gene *S*_*j*_

$$\begin{array}{c} \underset{\iota_{j},\tau_{j}}{\text{min}}E{\left(\zeta -\iota_{j}-\tau_{j}S_{j}\right)}^{2}. \end{array}$$ (15)

Let *α*_*j*_ and *β*_*j*_ be the solutions to Eq 15, and ${\hat{\beta }}_{j}$ be the least squares estimators for *β*_*j*_ for *j* = 1, …, *p*, where ${\hat{\beta }}_{j}={\left(S_{j}^{T}S_{j}\right)}^{-1}S_{j}^{T}\zeta$.

We assume the sample correlation between *S*_*j*_ and *S*_*k*_ is ${\hat{\rho }}_{jk}$, the sample standard deviation of *S*_*j*_ is ${\hat{\sigma }}_{jj}$, the conditional distribution of *ζ* given *S*_1_, …, *S*_*p*_ is $N(\mu \left(S_{1},\ldots ,S_{p}\right),\sigma^{2})$. The covariance of any two least squares estimators $\hat{\beta_{j}}$ and $\hat{\beta_{k}}$ is

$$\begin{array}{c} \text{Cov}\left(\hat{\beta_{j}},\hat{\beta_{k}}\right)=\text{Cov}\left(\sum_{i=1}^{n}\frac{S_{ij}}{{\hat{\sigma }}_{jj}^{2}}\zeta_{i},\sum_{i=1}^{n}\frac{S_{ik}}{{\hat{\sigma }}_{kk}^{2}}\zeta_{i}\right)=\sigma^{2}{\hat{\rho }}_{jk}/n{\hat{\sigma }}_{jj}{\hat{\sigma }}_{kk}. \end{array}$$ (16)

Furthermore, since *β*_*j*_ is the solution to Eq 15, $\beta_{j}=E\left({\left(S_{j}^{T}S_{j}\right)}^{-1}S_{j}^{T}\zeta \right)=E\left({\hat{\beta }}_{j}\right)$.

Therefore, the joint distribution of least squares estimators ${\hat{\beta }}_{1},\ldots ,{\hat{\beta }}_{p}$ is $N({\left(\beta_{1},\ldots ,\beta_{p}\right)}^{T},\Sigma^{*})$, where the (*j*, *k*)^*th*^ element of covariance matrix Σ* is $\Sigma_{jk}^{*}=\sigma^{2}{\hat{\rho }}_{jk}/n{\hat{\sigma }}_{jj}{\hat{\sigma }}_{kk}$.

Next, we standardize ${\hat{\beta }}_{1},\ldots ,{\hat{\beta }}_{p}$ using their standard deviations as suggested in [7]:

$$\begin{array}{c} {\hat{U}}_{j}=\frac{{\hat{\beta }}_{j}}{SD({\hat{\beta }}_{j})}=\frac{{\hat{\beta }}_{j}}{\sigma /(\sqrt{n}{\hat{\sigma }}_{jj})},\;j=1,\ldots ,p. \end{array}$$ (17)

Then, the joint distribution of standardized estimators ${\hat{U}}_{1},\ldots ,{\hat{U}}_{p}$ is $N({\left(\mu_{1},\ldots ,\mu_{p}\right)}^{T},\Sigma )$, where $\mu_{j}=\sqrt{n}\beta_{j}{\hat{\sigma }}_{jj}/\sigma$ for *j* = 1, …, *p* and the (*j*, *k*)^*th*^ element of covariance matrix **Σ** is $\Sigma_{jk}={\hat{\rho }}_{jk}$. It is obvious that if *S*_1_, …, *S*_*p*_ are correlated, then ${\hat{U}}_{1},\ldots ,{\hat{U}}_{p}$ are also correlated.

We can rewrite the standardized marginal regression estimators $\hat{U_{j}}$ as a model of true marginal regression coefficients and error terms of the marginal regression coefficients as the form in Eq 1

$$\begin{array}{c} {\hat{U}}_{j}=\mu_{j}+\upsilon_{j},\;j=1,\ldots ,p, \end{array}$$ (18)

where the true marginal genetic effects *μ*_*j*_ are assumed to follow a mixture distribution of a point mass at 0 (corresponding to no effect) and a nonparametric distribution (corresponding to having effects). The error terms of the standardized marginal regression coefficients *υ*_1_, …, *υ*_*p*_ follow a distribution of N(0,Σ). The covariance matrix **Σ** can have arbitrary dependent structure. We use empirical estimates ${\hat{\sigma }}^{2}$, ${\hat{\rho }}_{jk}$ and ${\hat{\sigma }}_{jj}$ to calculate the standardized estimators and **Σ**.

### Gene expression outcome and highly dependent covariance structure

From the gene expression data, we take the gene expressions of CHRNA6 (cholinergic receptor, nicotinic, alpha 6) as the outcome variable (*ζ*) in Eq 15. Because the gene CHRNA6 is known to be related to activation of dopamine releasing neurons with nicotine [21], it is a widely studied subject in nicotine addiction studies.

**Fig 4:**
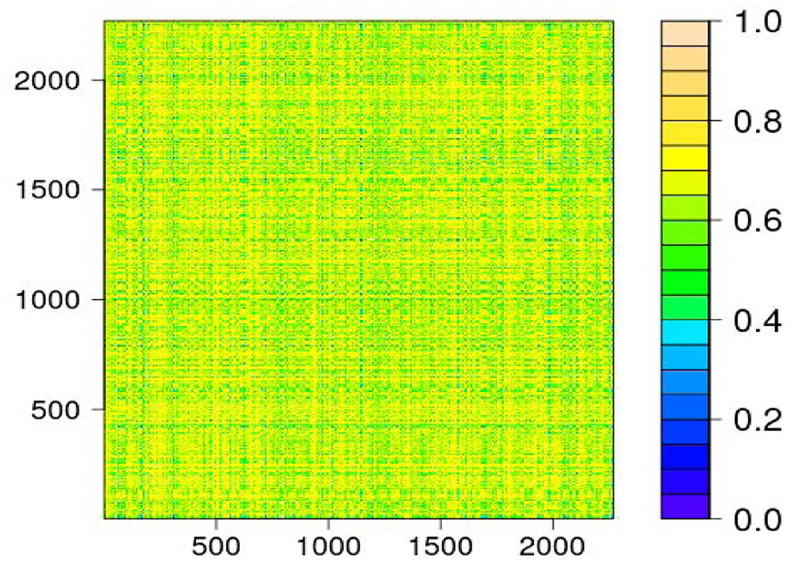
Heatmap of correlation between highly correlated genes.

In the remaining gene expressions, we use the method in [19] to first calculate correlations between a gene and all other genes, then count the number of correlations that are greater than 0.6 for this gene. If the count of >0.6 correlations for a gene is greater than 2, 200, we consider this gene as highly correlated with other genes. Therefore, there are 2, 269 genes satisfying this criterion. We take each of the 2, 269 genes as *S*_*j*_ in Eq 15, and store the gene data in the *n* × *p* matrix *S*, where *n* = 90, *p* = 2, 269. Fig 4 illustrates the heatmap of the absolute values of correlation among the 2, 269 genes.

Next, we regress CHRNA6 (*ζ*) on each of the 2, 269 gene expressions (*S*_*j*_). We use empirical estimates ${\hat{\sigma }}^{2}$, ${\hat{\rho }}_{jk}$ and ${\hat{\sigma }}_{jj}$ to calculate the standardized least squares regression coefficients (${\hat{U}}_{j}$), which have the distribution as shown in Eq 17. Lastly, using our proposed empirical Bayesian method (DepEB) described in Section, we obtain weakly dependent estimates and estimate the sparsity parameter and posterior distribution of the genes’ true marginal effect on CHRNA6. As a comparison, we also apply the EB estimation procedure proposed by [4] on the highly correlated gene data set.

### Analysis results

Our proposed DepEB estimation procedure considering dependence can identify 14 genes (0.617% of the studied 2, 269 genes) to be associated with CHRNA6, while the EB estimation procedure without considering dependence can not identify genes related to CHRNA6. Table 2 lists the gene names and the values of the posterior mean of the genes’ effects estimated by the DepEB procedure on CHRNA6.

**Table 2:** Genes associated with CHRNA6 estimated by the DepEB procedure.

Gene Name	Posterior Mean of the Gene’s effect on CHRNA6
GI_14149729-S	0.0403
**GI_14249217-S**	0.0886
**GI_19923528-S**	0.0011
GI_21314756-S	0.0002
GI_22907051-S	0.0002
**GI_32189368-S**	0.0034
**GI_32261328-S**	0.0002
GI_33239450-A	0.0002
GI_38327038-I	-0.0248
**GI_42659728-S**	0.0002
GI_4504190-S	0.0021
**GI_4506330-S**	-0.2308
GI_8922084-S	0.0003
**GI_9256536-S**	0.0019

Our findings are consistent with the findings in [19, 20], both of which also used the 90 JPT-CHB population’s gene data to identify significant genes associated with CHRNA6. Our proposed DepEB procedure selects genes GI_14249217-S, GI_19923528-S, GI_32189368-S, GI_32261328-S, GI_42659728-S, GI_4506330-S and GI_9256536-S, which are also among genes identified by [19] to be related to CHRNA6. In particular, [19] found that gene GI_42659728-S is very likely and gene GI_4506330-S is extremely likely to be related to the outcome CHRNA6. In addition, gene GI_32189368-S, i.e. POLE2, Homo sapiens polymerase (DNA directed), epsilon 2 (p59 subunit), was also discovered by [20] to be related to CHRNA6.

## Conclusion

In feature selection of normal mean problem, we propose a new DepEB estimator which extends the empirical Bayesian (EB) estimator method proposed by [4]. Our estimator allows arbitrary dependent structure in the error terms. We first apply eigenvalue decomposition to decompose correlated signals to common dependence and weakly dependent random errors. Next, we subtract the common dependence from the signals. We then get approximate likelihood of the sparse signals using the weakly dependent errors. The existence of sparsity makes the approximation feasible. Finally, we use an iterative maximization algorithm based on non-parametric kernel density to find the sparsity and the posterior distribution of the signals in a Bayesian estimating model.

In our simulation studies, we consider a number of covariance structures in relatively high sparse signals. The simulation results illustrate that when there exists moderate or strong correlations in the signals, our DepEB estimation procedure can correctly find the sparsity of the signals and produce a lower MSE compared to that in the EB estimation procedure ignoring the correlation structure in the sparse signals. Furthermore, we apply our proposed estimation procedure in the 90 JPT-CHB population’s gene expression data set with a highly dependent covariance structure. Our proposed DepEB estimation method outperforms the EB estimation method and identifies genes that are associated with the outcome gene CHRNA6. The findings in the real data analysis are consistent with those in other studies. Hence, this study provides an useful application for feature selection when facing strongly dependent covariates.

In the real data analysis of this study, we use marginal regression coefficients as the observed signals for the sparse vector. The reason of using marginal information is to deal with high dimensionality, see studies in [7, 22]. Therefore, one possible direction for future research is to use the appropriate initial estimators from the full regression model as the observed signals for sparse vector.
